# Initial experience with use of hydrogel microcoils in embolization of pulmonary arteriovenous malformations

**DOI:** 10.1186/2193-1801-3-609

**Published:** 2014-10-17

**Authors:** Keigo Osuga, Kentaro Kishimoto, Kaishu Tanaka, Masahisa Nakamura, Yusuke Ono, Noboru Maeda, Hiroki Higashihara, Tetsuro Nakazawa, Noriyuki Tomiyama

**Affiliations:** Department of Diagnostic and Interventional Radiology, Osaka University Graduate School of Medicine, 2-2 Yamadaoka, Suita, Osaka, 565-0871 Japan; Department of Diagnostic Radiology, Osaka Cardiovascular and Cancer Center, Osaka, Japan

**Keywords:** Pulmonary arteriovenous malformations, Embolization, Hydrogel microcoils

## Abstract

The purpose of this study is to describe our initial experience with embolization of pulmonary arteriovenous malformations (PAVMs) using hydrogel microcoils. The technical and radiological outcomes were retrospectively reviewed in seven patients with nine simple-type PAVMs (median feeder size 4 mm, range 3-6 mm) who underwent embolization. Hydrogel microcoils were mainly used, and detachable bare microcoils were combined as needed to occlude the terminal feeding artery just before the sac. Of a total of 43 microcoils, 30 (69.8%) hydrogel microcoils were deployed in eight PAVMs with the median number 3.5 (range 2 to 6) per lesion. All hydrogel microcoils were successfully deployed without microcatheter stuck or malposition. In the remaining one small PAVM, only soft bare microcoils were used, however, resulting in recanalization requiring additional coils in the second session. The venous sac was substantially shrunk in all lesions treated with hydrogel microcoils with the median size reduction rate 95.0% (range 81.8% to 99.0%) during the median follow-up period 10 months (range 6 to 18 months). In conclusion, hydrogel microcoils were safely and effectively applied for occluding PAVMs with relatively small feeders.

## Introduction

Pulmonary arteriovenous malformations (PAVMs) are abnormal fistulous connections between pulmonary arteries and veins forming a venous sac. PAVMs occur either sporadically or as a part of manifestations of hereditary hemorrhagic telangiectasia (HHT). The patients may suffer from stroke or brain abscess due to paradoxical embolism, dyspnea and fatigue due to hypoxemia, and rarely, hemoptysis or hemothorax due to spontaneous rupture of the venous sac. According to the international guidelines for the diagnosis and management of HHT, transcatheter embolization is the first-line treatment for symptomatic PAVMs (Faughnan et al. 2011). For asymptomatic PAVMs, the feeding artery 3 mm or greater in diameter is generally considered as the size threshold for embolization, although embolization can be also performed in smaller feeding arteries. Although efficacy and safety of coil embolization has been demonstrated in mostly case series, reperfusion of treated PAVMs mainly occur due to recanalization through the coils (Pollak et al. 2006). Therefore, it is important to increase the coil packing density to achieve high degree of cross-sectional occlusion for long-term occlusion (Pollak et al. 2006).

Recently, hydrogel coated detachable microcoils or hydrogel microcoils (Azur, Terumo, Tokyo, Japan) has been introduced for peripheral coil embolization (Nambiar et al. 2008). The hydrogel microcoils are coated with polymer that expand in contact with blood, and thus, have an advantage of greater filling volume compared with bare platinum coils. Although hydrogel microcoils theoretically lower the risk of recanalization, there have been few reports to describe details of their application in PAVM embolization. Therefore, the feasibility or role of hydrogel microcoils in this particular indication remains unclear. In this report, we describe our initial experience with use of hydrogel microcoils in a series of patients with PAVMs with relatively small feeding arteries.

## Materials and methods

### Patients

We retrospectively reviewed the medical records and radiological findings of seven consecutive patients with a total of nine PAVMs who underwent embolization mainly using hydrogel microcoils between December 2012 and June 2013. All patients were female, and the mean age was 57.9 years old (range 24 - 69). One patient had hereditary hemorrhagic telangiectasia (HHT), according to the Curaçao’s clinical diagnostic criteria (Shovlin et al. 2000). Six patients had a single PAVM, and one patient had three PAVMs. Four patients had past histories of neurological complications including brain infarction due to paradoxical embolism (n = 3) and spinal hemorrhage from spinal AVM (n = 1). All nine lesions consisted of a simple AV fistula with a single feeder and a single drainer. These feeding arteries were relatively small with the median diameter of 4 mm (range 3-6 mm). The median maximum size of venous sac was 13.5 mm (range 3 to 17 mm). Room air oxygen saturation was >95% in all patients, and none of them showed hypoxic symptoms. The indication for coil embolization included i) past history of paradoxical embolism (n = 3), ii) feeder size 3 mm or larger (n = 7), and/or iii) multiplicity (n = 1), that were considered as the further risks of paradoxical embolism. The written informed consent was obtained from in all patients, and this retrospective study was approved by the internal review board.

### Coils

Hydrogel microcoils are 0.018-inch detachable platinum microcoils coated with hydrogel polymer that expand when the polymer contact with blood. The hydrogel expands approximately five times the original volume of the bare coil, thus may offer more filling volume with potentially fewer number of microcoils. The available coil size were 3 mm, 4 mm, 5 mm, 6 mm, and 8 mm in loop diameter and 5 cm, 10 cm, and 20 cm in the extended length. The hydrogel microcoils were prepared and deployed according to the manufacturer’s instruction. For pre-softening, the stretched hydrogel microcoil was immersed in warm sterile saline at 60 degree C in five to ten seconds until the coil curls. When the hydrogel microcoil was appropriately positioned in the vessel, the coil was released with a detachment controller (V-Grip, Terumo, Tokyo, Japan). Repositioning time of the coil was limited within three minutes from the time of loading into the microcatheter, because it will become difficult to retract the coil once in contact with blood. It will take approximately 20 minutes until complete expansion of the hydrogel.

### Embolization procedures

All procedures were performed under local anesthesia. A 6Fr introducer sheath was inserted via the right femoral vein in six patients and via the right jugular vein in one patient because of lower leg paralysis and atrophy as a sequel of spinal hemorrhage. Diagnostic pulmonary angiography was performed using a 4Fr pigtail catheter sequentially to each lung to identify all lesions and their feeding arteries. Then, the pigtail catheter was exchanged to a coaxial guiding catheter systems (Medikit, Tokyo, Japan) consisting of an outer 6Fr multipurpose guiding catheter and an inner 4Fr catheter with a short distal angle. Following the selective insertion of the inner coaxial catheter into the feeding artery, the outer guiding catheter was advanced over it to provide adequate support for coil deployment maneuvers. When the coaxial guiding catheter system was settled, a 2.0Fr two-marker microcatheter with inner diameter 0.022 inch (Progreatβ^3^, Terumo, Tokyo, Japan) was coaxially advanced in the distal feeding artery as close to the venous sac as possible. As a rule, the terminal segment of the feeding artery beyond any significant normal branches was occluded with mainly hydrogel microcoils. Because of the limited reposition time of the hydrogel microcoil within three minutes, the attending nurse measured the time from the coil loading into the microcatheter, and called how much time was left per minute to complete the coil deployment. Softer bare platinum detachable microcoils (Helipaq or Cashmere, Codman & Shurtleff, Raynham, MA, USA) were combined as needed. For the first coil, an oversized coil was deployed to avoid coil migration into the pulmonary vein. If there was a small distal normal branch near the venous sac, the anchor technique was applied to secure the coil stability, where the initial few centimeters of the coil was hooked in the side branch and the rest of the coil was released in the feeding artery. Once the first coil was settled, hydrogel microcoils were mainly used for the filling of the feeding artery until the blood flow substantially reduced by test injection of contrast media. The hand injection angiography was sequentially obtained 10 minutes and 20 minutes after the last hydrogel microcoil was deployed. If there was residual blood flow even after 20 minutes, additional hydrogel coils or softer bare platinum microcoils were deployed as needed. Finally, adequate occlusion of the feeding artery was confirmed by the main pulmonary angiography, and it was also assessed if any accessory feeding artery was missed. Technical success was defined as complete occlusion of the targeted lesions at the end of procedure.

During the procedure, patients received prophylactic antibiotics with 1 g of intravenous cefazolin sodium. They also received 3,000 – 5,000 U of intravenous heparin to maintain the activated clotting time (ACT) beyond 200 seconds to avoid the thrombi formation around the catheters and coils that could cause paradoxical embolism before the complete occlusion was achieved.

### Image assessment

Follow-up CT scans using 64-channel multi-detector CT scanners (LightSpeed VCT, GE Healthcare, Milwaukee, WI, USA) were performed between 6 months and 18 months after the procedure to evaluate the shrinkage of venous sac and any coil-associated complication. The whole lung non-contrast CT images were obtained with a 0.625 mm collimation and 1.375 pitch. Efficacy of embolotherapy was assessed by the bi-dimensional size reduction rate of the venous sac at its longest diameter on the follow-up axial CT images in the lung window setting. The measurement of the venous sac was performed by consensus of the two board-certified diagnostic radiologists from authors.

## Results

**Figure 1:**
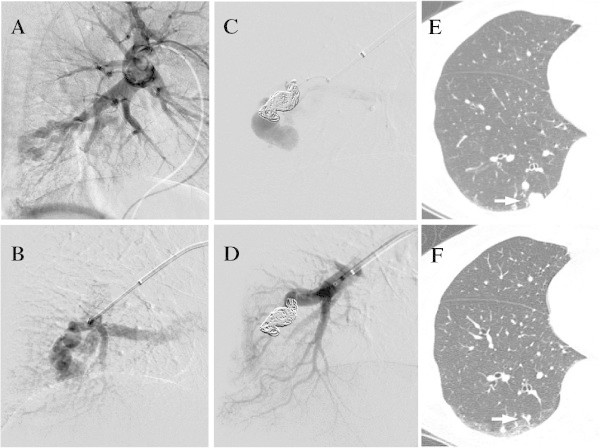
**A 66 year-old woman with the right PAVM (Case 2). A**. The pulmonary arteriogram (lateral projection) shows a simple type AVM of the posterior segment. **B**. The selective angiogram shows the tortuosity of the terminal feeding artery beyond the normal branch. **C**. A microcatheter was advanced as close to as possible to the sac. Following the two oversized 10 mm and 6 mm anchoring bare microcoils, four 6-3 mm hydrogel microcoils were deployed for filling a short segment of feeding artery. Immediately after embolization, a minimal flow remained in the venous sac. **D**. Post embolization angiogram at 20 minutes shows the complete PAVM occlusion. **E**. The baseline non-contrast CT image shows the venous sac on the posterior lung surface (arrow). **F**. The six-month follow-up CT image shows shrinkage of the venous sac (arrow).

All embolization procedures were successfully completed. The technical and radiological results are summarized in Table 1. Of a total of 43 microcoils, 30 (69.8%) hyrdogel microcoils were deployed in eight PAVMs with the median number 3.5 (range 2 to 6) per lesion. All hydrogel microcoils were deployed within one to two minutes. Neither coil stuck in the microcatheter, malposition in the target vessel, nor migration into non-target vessels occurred. Exceptionally, in one small PAVM, only soft bare platinum microcoils were deployed because of the microcatheter instability in the small and tortuous feeding artery (Case 6, lesion no. 8). In five PAVMs, oversized bare platinum microcoils were combined for the first one or two coils to create a scaffold for the following hydrogel microcoils. In two PAVMs, small softer bare platinum microcoils were added proximal to the hydrogel microcoils. The ratio of number of hydrogel microcoils per total number of coils ranged from 42.9% to 100% with the median 70.9%. The ratio of total length of hydrogel microcoils per the total length of all coils ranged from 41.7% to 100% with the median 66.7%. Complete feeder occlusion was observed at 10 minutes in two lesions (25%), and at 20 minutes at three lesions (37.5%) (Figure 1). In the remaining three PAVMs (37.5%), additional hydrogel microcoils (n = 2) or softer bare platinum microcoils (n = 1) were added because of incomplete occlusion at 20 minutes.

**Figure 2:**
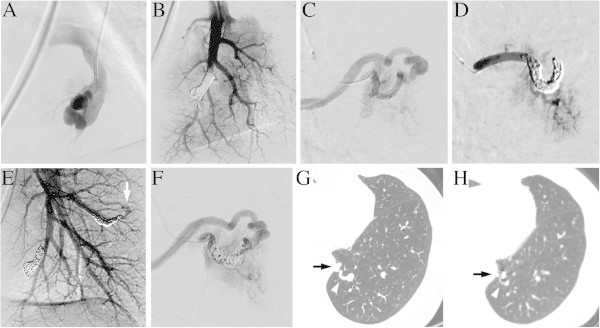
**A 62 year-old woman with three PAVMs (Case 6). A**. The selective angiogram shows the PAVM in the posterior segment. **B**. The distal feeding artery was embolized with three 6-4 mm hydrogel microcoils and four additional 3 mm bare platinum microcoils. **C**. Selective angiogram of the small PAVM in the superior segment shows the tortuosity of the distal feeding artery. **D**. Because of microcatheter instability, six 4-3 mm 0.014-inch bare detachable microcoils alone were deployed. Post embolization angiogram shows the feeding artery occlusion. **E**. After two months, the follow-up pulmonary arteriogram was obtained in the second session to treat the PAVM in the right lung (not shown). The PAVM in the posterior segment treated by hydrogel microcoils remained occluded, whereas the smaller PAVM in the superior segment treated by bare microcoils alone shows recanalization (arrow). **F**. Selective angiogram reveals the recanalization through the coil interstices. Four additional 3 mm bare microcoils were packed proximally to achieve complete occlusion (not shown). **G**. The baseline non-contrast CT image shows the venous sac (arrow) and the draining vein of the PAVM in the posterior segment (arrowhead). **H**. The eleven-month follow-up CT shows shrinkage of the venous sac (arrow) and normalization of the draining vein size (arrowhead).

In one patient with three PAVMs, the two lesions of the left lung and the one lesion of the right lung were treated in two separate sessions with two-months interval. In the second session, the left pulmonary angiography revealed that the lesion treated by hydrogel microcoils was completely occluded, whereas the other smaller lesion treated by bare microcoils alone showed recanalization of the feeding artery through the coil interstices (Figure 2). Therefore, small bare platinum microcoils were added proximal to the previous coils. The venous sac was substantially shrunk in all PAVMs treated with hydrogel coils. The median size reduction rate of venous sac was 95.0% (range 81.8 to 99.0%) during the median follow-up period 10 months (range 6 to 18 months). During the follow-up, no minor or major complication was observed in all patients.

**Table 1:** **Summary of the technical and radiological results**

Case no./PAVM no.	Feeder diameter (mm)	No. of hydrogel microcoils (%)	Total length (cm) of hydrogel microcoils (%)	Loop size of hydrogel microcoils	Venous sac size (baseline)	Venous sac size (last follow-up)	Follow-up periods (months)	Size reduction rate (%)
1-1	6	6 / 6 (100)	60 (100)	8-3	14 × 10	7 × 1	12	95.0
2-2	5	4 / 6 (66.7)	40 (42.5)	6-3	17 × 10	4 × 2	6	95.3
3-3	3	3 / 4 (75.0)	25 (55.6)	4-3	3 × 3	1 × 1	6	88.9
4-4	4	5 / 5 (100)	60 (100)	6-3	16 × 12	2 × 1	6	99.0
5-5	3	2 / 3 (66.7)	40 (66.7)	4	14 × 9	3 × 2	14	95.2
6-6	4	3 / 7 (42.9)	60 (75.0)	6-4	13 × 7	3 × 2	11	93.4
6-7	3	3 / 7 (42.9)	30 (41.7)	4-3	11 × 6	4 × 3	9	81.8
6-8*	3	0 / 10 (0)	0 / 56 (0)	NA	8 × 5	4 × 3	9	70.0
7-9	5	4 / 5 (80)	60 (66.7)	4-3	11 × 10	2 × 2	6	96.4

* Only bare microcoils were used in two sessions. NA: not applicable.

## Discussion

Transcatheter embolization has been the treatment of choice for occluding PAVMs. Fibered pushable coils have been widely used for the mechanical occlusion of feeding arteries (Pollak et al. 2006). Recently, the Amplatzer vascular plug (AVP) family has been also introduced to occlude large feeders safely with a single device or in combination with coils (Trerotola & Pyeritz 2010). On the other hand, the advance in microcoils and microcatheter technology has enabled selective embolization of small or tortuous vessels, where insertion of the regular delivery catheters is considered difficult or harmful. Especially, soft detachable microcoils, although they are expensive, have been increasingly applied in PAVM embolization, because they can be repositioned, offer more precise deployment, and prevent systemic coil migration (Dinkel & Triller 2002; Greben et al. 2013). However, such soft and thin bare platinum detachable microcoils are susceptible for recanalization. Indeed, in our series, one small lesion treated by bare platinum microcoils alone showed early recanalization in two months. In the literatures, recanalization after successful embolization has been the main cause for reperfusion of PAVMs (Pollak et al. 2006; Milic et al. 2005). The reported recanalization rates were up to 20% of PAVMs treated with coils (Pollak et al. 2006; Milic et al. 2005) and 5 to 10% of PAVMs treated with AVPs (Trerotola & Pyeritz 2010; Fidelman et al. 2008), possibly through the coil interstices or plug meshes. The technical reason for recanalization may be inadequate cross-sectional occlusion with a fewer number of devices or those of inappropriate size than desired (Milic et al. 2005). Therefore, dense cross-sectional occlusion by filling the coil interstices is one of the solutions to enhance the packing density.

The main advantage of hydrogel microcoils is to increase the packing density by hydrogel expansion among the coil interstices and to establish tight mechanical vessel occlusion without the aid of thrombus formation. These features may be helpful in the embolization procedure like for PAVMs under systemic heparinization. However, there have been few reports describing application of hydrogel microcoils for PAVM embolization. In an early report of peripheral application of hydrogel microcoils, the coils were deployed through the existing coil mesh in a partially recanalized PAVM (Nambiar et al. 2008). The additional increase in volume after hydrogel swelling helped to achieve complete occlusion of the PAVM. In other reported indications, hydrogel microcoils reduced the coil numbers when compared with fibered microcoils in prophylactic embolization of the gastroduodenal artery before yttrium-90 radioembolization for liver cancers (Maleux et al. 2013). In another comparison study for intracranial aneurysms, the higher packing density, shorter total coil length, and lower recanalization rates were proven in the hydrogel microcoil group than in the bare coil group (White et al. 2011). The study suggested that hydrogel microcoils constitute at least 50% of the total coil length for <10 mm aneurysms, and at least two thirds of the total coil length for >10 mm aneurysms. In our series, the median proportion of hydrogel microcoils was 66.7% of the total coil length. However, there has been no report yet about the optimal proportion of hydrogel microcoils to decrease the recanalization rates of PAVMs. Although the significant sac shrinkage was obtained in our series in the short period up to eighteen months, longer-term follow-up is necessary to ensure the sac involution or freedom from symptoms, as reperfusion can occur in once resolved PAVMs even after more than two years (Milic et al. 2005).

There were some disadvantages in the hydrogel coils. We occasionally experienced slight resistance in the coil delivery, and the microcatheter kicking-back, although no coil resulted in the catheter stuck or malposition in the target vessel. Because of the relatively high coil rigidity, adequate support by a deeply inserted guiding catheter and the use of braided microcatheter was important for controlled delivery of the hydrogel microcoils. High-flow type microcatheters with inner-diameter 0.027 inch would be also helpful for better passage of the hydrogel microcoils. In addition, the limited reposition time within three minutes was stressful for operators, but all hydrogel microcoils could be deployed within two minutes that allowed one or two times attempts of coil reposition. In our early experience, we waited for full hydrogel expansion up to 20 minutes after the last hydrogel coil was deployed. The progressive vessel occlusion was observed in five of eight lesions even under systemic heparinization.

There are several limitations in our study. First, this is a retrospective short-term observational study with a small number of patients. Second, only 0.018-inch hydrogel microcoils were evaluated in PAVMs with relatively small feeding arteries, as 0.035-inch hydrogel coils were not yet available in our country. Third, contrast-enhanced CT scan was not performed in our series, thus reperfusion of PAVM could not be precisely assessed. However, the marked shrinkage of venous sac was good indicator for efficacy of embolization even on plain CT images. Forth, it still remains unclear if combining the hydrogel microcoils can reduce the total coil number or the recanalization rates in PAVM embolization. Cost-effectiveness should be also considered, as the hydrogel microcoils are more expensive than other coils in our country. Therefore, a further study would be necessary to prove the true advantage of combining hydrogel microcoils over conventional coils alone by a randomized controlled trial. For larger feeding arteries where the regular guiding-coaxial catheters are accessible, the terminal feeder occlusion using 0.035-inch fibered coils with or without combining AVPs may remain the suitable procedure.

In conclusion, the hydrogel microcoils were safely and effectively applied for occluding PAVMs with relatively small feeders. The substantial sac shrinkage was obtained in all treated PAVMs. Further study will be needed to confirm if hydrogel microcoils reduce the recanalization rates.
